# Artificial Intelligence in Cardiac Point-of-Care Ultrasound: A Narrative Review

**DOI:** 10.3390/diagnostics16121921

**Published:** 2026-06-21

**Authors:** Evan Avraham Alpert, Toby Kwartz, Barry Hahn, Waid Abdulghani, Ahmad Nama, Ziv Dadon

**Affiliations:** 1 Department of Emergency Medicine, Hadassah University Hospital-Ein Kerem, Jerusalem 91120, Israel; 2 Faculty of Medicine, Hebrew University of Jerusalem, Jerusalem 91905, Israel; 3 Department of Emergency Medicine, Northwell, New Hyde Park, New York, NY 11040, USA; 4 Department of Emergency Medicine, Staten Island University Hospital, Staten Island, NY 10305, USA; 5 Cardiology Department, Jesselson Integrated Heart Center, Eisenberg R&D Authority, Shaare Zedek Medical Center, Jerusalem 91031, Israel

**Keywords:** artificial intelligence, echocardiography, emergency medicine, point-of-care systems, ultrasonography

## Abstract

**Background**: Cardiac point-of-care ultrasound (POCUS) is widely used in emergency and acute care settings. Still, broader use remains limited by operator dependence and variability in image acquisition and interpretation. Artificial intelligence (AI), including machine learning and deep learning methods, has been applied to cardiac POCUS to support image acquisition, automate quantitative measurements, and assist interpretation. **Methods**: We performed a narrative review of current applications of AI-assisted cardiac POCUS. A targeted literature search of PubMed and Google Scholar from 2018 to 2026 was conducted using terms related to AI, machine learning, deep learning, and cardiac ultrasound. Studies evaluating AI-assisted cardiac ultrasound in clinical, educational, or image-acquisition settings were included, with emphasis on recent, clinically relevant applications. **Results**: The most developed application of AI-assisted cardiac POCUS is an automated assessment of left ventricular systolic function, particularly the left ventricular ejection fraction (LVEF), where multiple studies report agreement with expert interpretation or formal echocardiography and improved performance among novice users. AI-assisted tools have also been evaluated for pericardial effusion detection, guidance for image acquisition, and education. More complex applications, including diastolic function assessment and hemodynamic measurements such as LVOT-VTI, remain less well validated and more dependent on image quality. Across studies, performance is closely linked to image acquisition quality and has often been evaluated under controlled rather than real-world conditions. **Conclusions**: Current evidence supports AI-assisted cardiac POCUS primarily as a decision-support tool, with the strongest data for automated assessment of LVEF. Other applications remain investigational.

## 1. Introduction

Cardiac point-of-care ultrasound (POCUS) began more than 20 years ago in emergency medicine, initially as a focused bedside tool to assess cardiac motion and identify pericardial effusion. Since then, its use has expanded across multiple specialties, including critical care, internal medicine, family medicine, and pediatrics [1,2,3,4]. Across these disciplines, the applications of cardiac POCUS have also broadened, with growing emphasis on the rapid bedside assessment of shock, presence of pericardial effusion, ventricular systolic function, and other important cardiac pathologies [5,6,7].

Despite these advances, achieving consistent diagnostic accuracy remains challenging and is often dependent on operator experience and training. Variability in image acquisition and interpretation continues to limit broader adoption, particularly among less experienced users [8]. These limitations are especially relevant in emergency and acute care environments, where examinations are often performed under time pressure, with suboptimal acoustic windows, limited patient cooperation, positioning challenges, and operators with varying levels of ultrasound expertise.

Artificial intelligence (AI), including machine learning and deep learning, has emerged as one possible approach to addressing some of these limitations. In the context of cardiac POCUS, AI systems are being developed to assist with image acquisition, automate quantitative measurements, and support interpretation by identifying patterns within ultrasound data that may not be readily recognized during bedside assessment alone [9,10,11,12,13,14]. These applications are particularly appealing in cardiac POCUS because they target some of the most operator-dependent parts of the examination, including obtaining interpretable views, estimating left ventricular systolic function, and performing more structured measurements.

At the same time, the clinical role of AI-assisted cardiac POCUS remains incompletely defined. The current literature includes a broad range of use cases, from real-time acquisition guidance and automated left ventricular ejection fraction (LVEF) assessment to educational support and identification of specific cardiac pathologies. However, these applications differ noticeably in technical maturity, reference standards, operator populations, operational simplicity, and clinical readiness. As a result, AI-assisted cardiac POCUS should not be viewed as a single, uniform intervention, but rather as a set of related tools with varying levels of supporting evidence and potential roles in bedside care.

Importantly, AI-assisted cardiac POCUS should not be viewed as a single intervention. Current applications include acquisition guidance, automated quantification, diagnostic classification, hemodynamic measurement, and educational support. The literature also includes different implementation circumstances, including AI applied to comprehensive echocardiographic workflows, AI interpretation of stored or expert-acquired cardiac ultrasound clips, real-time handheld or bedside POCUS interpretation, and AI acquisition guidance for novice or nonexpert users. These applications differ in their technical requirements, reference standards, operator dependence, and likely clinical roles. For example, automated LVEF estimation is a different task than guiding a novice user toward an interpretable view, detecting pericardial effusion, estimating left ventricular outflow tract velocity–time integral (LVOT-VTI), or providing feedback during training. For this reason, the evidence for one AI application should not be assumed to apply to another.

The objective of this narrative review is to describe the current applications of AI-assisted cardiac POCUS and to evaluate its role in augmenting diagnostic accuracy, clinical decision-making, and education.

## 2. Methods

This narrative review was conducted to evaluate current applications of AI in cardiac POCUS. A targeted literature search was performed using PubMed and Google Scholar for studies published between January 2018 and March 2026. Search terms included “artificial intelligence,” “machine learning,” “deep learning,” “point-of-care ultrasound,” “cardiac ultrasound,” “focused cardiac ultrasound,” and “echocardiography.” Additional relevant studies were identified through a review of references from selected articles.

Studies were included if they evaluated AI-assisted cardiac ultrasound in clinical, educational, image-acquisition, automated-measurement, or diagnostic-support settings. Studies focused primarily on non-cardiac ultrasound, non-AI-assisted ultrasound, or non-English publications were excluded.

Studies were selected based on relevance to predefined domains of interest, including image acquisition guidance, automated quantification, diagnostic support, hemodynamic measurement, educational use, and detection of specific cardiac pathologies. Greater emphasis was placed on studies with direct relevance to bedside cardiac POCUS, including studies involving real-time AI integration, handheld ultrasound devices, emergency or acute care settings, nonexpert or bedside operators, clinically relevant reference standards, and outcomes beyond technical feasibility when available. Studies focused mainly on comprehensive echocardiography or purely technical model development were used only when they provided relevant background or context for cardiac POCUS applications.

Because this was a narrative rather than a systematic review, formal duplicate screening was not performed, and no formal PRISMA workflow or risk-of-bias instrument was used. Screening and study selection were based on author review and expert judgment. When uncertainty arose regarding study inclusion or interpretation, disagreements were resolved through discussion and consensus among the authors.

The heterogeneity of study designs, reference standards, operator experience, ultrasound platforms, and study endpoints was addressed qualitatively in the synthesis and interpretation of the literature. Studies were summarized with emphasis on clinical applicability, technical maturity, external validation, and relevance to emergency and acute care practice.

### 2.1. Technological Aspects of AI-Assisted Cardiac POCUS

AI applications in cardiac POCUS are primarily driven by machine learning and deep learning techniques. Machine learning algorithms are trained on large datasets to recognize patterns and perform tasks such as cardiac chamber identification and automated parameter measurement, including LVEF. Deep learning, a subset of machine learning, uses neural networks to analyze more complex imaging data and can support both image interpretation and classification [9,10,11,12,13,14].

In cardiac POCUS, AI applications can be broadly grouped into three domains: image acquisition guidance, automated quantification, and diagnostic support. Real-time guidance systems may help operators obtain interpretable views by providing feedback on probe positioning. At the same time, automated analysis tools can calculate parameters such as LVEF and the LVOT-VTI. Other systems can classify image quality and identify abnormalities in cardiac function [10,11,12,13,14,15].

These developments may reduce operator dependence and expand the use of cardiac POCUS in settings where advanced echocardiographic expertise or high-end devices are not readily available. However, most current systems remain dependent on adequate image acquisition, and performance in controlled study settings may not fully reflect real-world bedside use. This is particularly relevant in the emergency department (ED) and ICU, where limited time, patient instability, and suboptimal acoustic windows may challenge both human operators and AI systems [10,11,12,13,14,15].

Because LVEF remains central to the diagnosis, classification, management, and prognosis of heart failure, AI-assisted LVEF assessment has become the most developed application in this area [16]. Among current applications, the strongest evidence is for automated assessment of left ventricular systolic function. Table 1 summarizes selected representative studies of AI-based applications in cardiac POCUS, including image acquisition guidance, automated quantification, and diagnostic support.

The regulatory and commercial landscape is also changing. Several AI-enabled echocardiographic and handheld ultrasound tools now provide acquisition guidance or automated quantification. However, regulatory clearance does not mean broader clinical validation across emergency and critical care settings. It also does not resolve questions of clinical accountability. For bedside users, the more appropriate question is whether performance remains reliable across heterogeneous patients, operators, and clinical workflows, and how AI output should be documented, verified, and acted upon in real-time clinical care.

### 2.2. AI-Assisted Assessment of Left Ventricular Systolic Function and Heart Failure

LVEF is a central measure of left ventricular systolic function and remains an important marker for the diagnosis, classification, management, and prognosis of heart failure [16]. AI-assisted cardiac POCUS can rapidly calculate LVEF, aiding physicians in the ED, ICU, and other critical care settings who may not have the expertise to estimate it solely from cardiac ultrasound [17].

Luong et al. assessed the accuracy of AI-derived LVEF compared with expert clinicians.

Seven physicians and two nurse practitioners, all with expertise in heart failure and advanced echocardiography training (level 2), acquired the images. Echocardiographic clips were imported into a database and analyzed by an AI-assisted model that estimated LVEF and provided a qualitative assessment of image quality, classifying studies as poor, moderate, good, or excellent. The study showed that AI can estimate LVEF with reported agreement under controlled acquisition conditions. AI-derived estimates showed strong agreement with both expert interpretation and formal echocardiographic reports, demonstrating the AI’s ability to calculate LVEF under favorable conditions [17]. However, the model was evaluated offline after image acquisition rather than in real time at the bedside. In addition, performance was highly dependent on image quality. Of 1257 echocardiographic clips in the dataset, 341 were analyzable by the AI program, whereas 1096 were interpretable by an expert echocardiographer (level 3). These groups were not mutually exclusive [17]. These findings support AI-assisted interpretation under controlled conditions, while also showing that performance depends on analyzable image quality.

Tromp et al. evaluated the extent to which AI-assisted cardiac POCUS could balance diagnostic accuracy with ease of use in a setting involving practitioners with relatively limited echocardiographic skills. This study assessed the effectiveness of AI-assisted POCUS in identifying signs of heart failure. The two diagnostic parameters measured were LVEF and left atrial volume index, the latter being an echocardiographic marker associated with elevated left ventricular end-diastolic pressure. Seven nurses participated in the study as the practitioners performing the cardiac ultrasounds. The nurses underwent one day of training at the start of the study and another session one month later to ensure that their ultrasonography skills were sufficient. Nurses performed cardiac POCUS examinations during at-home outpatient visits in 94 patients. The examinations were assessed offline for LVEF and left atrial volume index using an AI-assisted algorithm. The patients subsequently underwent conventional transthoracic echocardiography (TTE) in the clinic, and measurements from the formal study served as the reference standard. The AI model demonstrated acceptable performance in assessing LVEF. Quantitative performance metrics for the left atrial volume index were not reported in comparable detail, limiting the interpretation of this component of the model [18]. This study is important because it extends AI-assisted cardiac POCUS beyond expert or near-expert users and into a pragmatic screening setting. At the same time, analyzable image quality remained an important constraint. From a clinical standpoint, the study is more persuasive as evidence of screening feasibility than as proof that AI can independently diagnose heart failure.

Dos Santos et al. further evaluated the role of AI-assisted POCUS in identifying heart failure and facilitating earlier initiation of care in primary care settings. In this study, patients older than 50 years with suspected heart failure were assigned to one of four groups: standard of care alone, standard of care plus NT-proBNP, standard of care plus NT-proBNP and AI-POCUS, and standard of care plus AI-POCUS. The primary outcomes included the rate of new heart failure diagnosis, initiation of guideline-directed treatment, and improvement in health-related quality of life [19]. This study is significant because it moves beyond image interpretation toward workflow- and patient-level applications with clinical outcomes. It suggests that AI-assisted cardiac POCUS may support earlier recognition of cardiac dysfunction in outpatient settings. However, the study does not establish that AI alone was responsible for the observed improvement in clinical performance.

LVEF may also be used for prognostic purposes to assess overall cardiac function and reduced systolic performance [16]. Cheema et al. described the use of AI-assisted cardiac POCUS in critically ill ICU patients during the COVID-19 pandemic, demonstrating how automated measurements and real-time guidance could assist clinicians in identifying ventricular dysfunction and hemodynamic abnormalities at the bedside [20]. Although this report was based on a small series, it shows how AI could be integrated into bedside decision-making in high-acuity settings.

Similarly, Dadon et al. prospectively evaluated the accuracy of an online AI-assisted tool for LVEF assessment using a handheld ultrasound device among patients with COVID-19, as compared with offline expert echocardiographer assessment of the same acquired clips. High correlation and substantial agreement were shown between the two assessments, and AI-assisted identification of reduced systolic function predicted worse composite outcomes [21]. These findings suggest that AI-assisted algorithms integrated into handheld ultrasound devices may function as real-time decision-support tools for automatic LVEF assessment and risk stratification.

A prospective study by Dadon et al. examined the ability of nine fourth- to sixth-year medical students to quantify LVEF during handheld ultrasound-acquired cardiac POCUS among 88 patients admitted to a cardiology ward, with and without blinded online AI assistance. The students completed a six-hour course that included preliminary testing, lectures, and practical training, during which each student performed at least four supervised cardiac POCUS examinations, including LVEF assessment. The patients subsequently underwent formal TTE, and student assessments (using the handheld device) were compared with those of board-certified cardiologists interpreting the same handheld ultrasound clips and with those of fellowship-trained echocardiographers interpreting the formal study (using the high-end device). This study demonstrated the benefit of real-time AI assistance in cardiac POCUS performed by novice users for LVEF assessment. Of the 88 patients included, the AI tool calculated LVEF for 82 patients. Without AI assistance, the students’ assessments showed only moderate correlation with cardiologist interpretations, while using the AI tool substantially improved the correlation. Comparison with formal echocardiography showed similar substantial agreement for student-plus-AI assessment and cardiologist interpretation, whereas student visual assessment alone had only fair agreement [22]. These findings are potentially clinically relevant because they suggest that AI may be particularly useful where human visual estimation is weakest, specifically among novice users and operators with limited experience.

In a subsequent follow-up study from the same registry, reduced systolic function based on student-plus-AI assessment independently predicted one-year mortality and cardiovascular-related readmission and was associated with unfavorable in-hospital outcomes [23]. Additional recent studies and reviews have further supported AI-assisted LVEF assessment using handheld ultrasound devices, while emphasizing the need for broader validation across patient populations, operators, and reference standards [24,32].

### 2.3. Detection of Pericardial Effusion

AI-assisted tools may also enhance the detection and localization of pericardial effusion on cardiac POCUS, particularly in emergency settings and during rapid-assessment examinations. Prompt identification of pericardial effusion is potentially clinically relevant because it may indicate cardiac tamponade necessitating immediate management [33]. This application was evaluated by Yıldız Potter et al., who developed and tested an AI algorithm for automated detection and localization of pericardial effusion from cardiac POCUS examinations. The study used 37 positive POCUS clips with pericardial effusion and 39 negative controls. The model tested offline demonstrated strong diagnostic performance, with a sensitivity of 89%, specificity of 91%, and overall accuracy of 92%. In addition to distinguishing between positive and negative cases, the algorithm also localized the effusion within the image [25].

This is an attractive emergency medicine application because it is relatively binary, time-sensitive, and clinically meaningful. The study supports the feasibility of AI-assisted analysis for detecting pericardial effusion on cardiac POCUS. However, the dataset was small, and broader clinical implementation would require validation in more diverse real-world populations. More recent primary care AI-POCUS data also suggest that pericardial effusion detection may be one of the triage-level cardiac tasks that can translate reasonably well into broader clinical use [34].

Taken together, these limited findings suggest that AI-assisted detection of pericardial effusion represents a relatively well-suited early use case for bedside implementation, given the binary nature of the diagnosis and its clinical urgency. However, broader validation in heterogeneous patient populations remains necessary.

### 2.4. Inferior Vena Cava Assessment

Inferior vena cava (IVC) collapsibility is commonly used as a bedside ultrasonographic parameter to assess intravascular volume status and fluid responsiveness, although its interpretation remains imperfect and highly context-dependent. Respiratory variation in IVC diameter can provide a rapid noninvasive estimate of hemodynamic status. Still, bedside use may oversimplify a more complex physiological question, and patient factors, operator experience, and image acquisition quality may influence interpretation [35].

Blaivas et al. investigated the accuracy of an AI-based deep learning model for determining IVC collapsibility from point-of-care ultrasound clips. The model was trained on 220 publicly available proximal IVC videos and then tested on 50 clips that were also independently reviewed by three blinded ultrasound experts. The AI model demonstrated moderate agreement with expert assessment and showed reasonable performance in classifying IVC collapsibility above or below 25% [26].

These findings suggest that AI-assisted IVC assessment may help standardize the interpretation of a commonly used operator-dependent bedside measurement. However, even with accurate automated measurement, the physiologic relationship between IVC collapsibility and true intravascular volume status remains limited. Factors such as mechanical ventilation, intra-abdominal pressure, right heart function, respiratory effort, and patient-specific physiology can all influence inferior vena cava dynamics and limit the reliability of collapsibility as a surrogate for fluid responsiveness [35].

As a result, the clinical value of AI-assisted IVC assessment is constrained not only by measurement variability but also by the underlying limitations of the parameter itself. Even if AI improves measurement consistency, IVC collapsibility remains an imperfect surrogate for intravascular volume status and fluid responsiveness. Therefore, AI may standardize how IVC collapsibility is measured, but it does not make the parameter itself physiologically definitive.

Clinicians should also recognize situations in which AI-assisted IVC assessment may be misleading. Automated measurement may appear precise even when the underlying physiologic interpretation is unreliable. This is especially relevant in mechanically ventilated patients, patients with marked spontaneous respiratory effort, elevated intra-abdominal pressure, right ventricular dysfunction, abnormal venous compliance, or other conditions that alter venous return and IVC dynamics. In these settings, AI may standardize the measurement but cannot determine whether IVC collapsibility accurately reflects fluid responsiveness. AI-assisted IVC assessment should therefore be interpreted as one component of hemodynamic assessment rather than as an isolated trigger for fluid administration or withholding fluids.

### 2.5. AI-Assisted Assessment of Diastolic Function

While most AI applications in cardiac POCUS have focused on systolic function, emerging studies have also evaluated their role in assessing diastolic function. In clinical practice, diastolic dysfunction is more complex to assess at the bedside and generally requires integration of multiple echocardiographic parameters, including Doppler assessment. For that reason, this is a particularly important test of whether AI can extend cardiac POCUS beyond simple visual estimation.

Gottlieb et al. evaluated the diagnostic accuracy of AI for identifying systolic and diastolic cardiac dysfunction in the ED. In this study, emergency physicians with ultrasound fellowship training received a brief training session on using the integrated AI tool on a handheld ultrasound device. The AI model provided live feedback and automated measurements used to assess cardiac function. Systolic function was evaluated using automated LVEF measurements, whereas diastolic function was assessed using mitral inflow and tissue Doppler-based parameters together with left atrial volume. When compared with expert assessment, the AI model demonstrated high sensitivity and specificity for both systolic and diastolic dysfunction [27]. Because systolic function is addressed elsewhere in this review, the main relevance of this study is that it extends AI-assisted cardiac POCUS beyond automated left ventricular ejection fraction assessment alone and suggests that AI may also support bedside evaluation of diastolic dysfunction. This is particularly important because diastolic function is more difficult to assess visually and usually requires a more structured echocardiographic approach. At the same time, the study compared AI-assisted measurements with expert interpretation rather than with a complete formal TTE as the reference standard [27]. Although the findings are encouraging, they should be interpreted cautiously. Diastolic function is difficult to assess at the bedside and depends on multiple Doppler-derived variables, loading conditions, rhythm status, and clinical context. Therefore, these data support feasibility, but they do not establish that AI-assisted cardiac POCUS can reliably diagnose diastolic dysfunction or replace comprehensive echocardiography. This application remains less mature than automated LVEF assessment.

### 2.6. Automated Left Ventricular Outflow Tract Velocity–Time Integral Measurement

In emergency and critical care settings, measurement of the LVOT-VTI can provide useful noninvasive information about stroke volume and cardiac output, which are especially important in the setting of undifferentiated shock, suspected cardiogenic shock, and fluid responsiveness assessment. Because LVOT-VTI is technically more difficult to obtain and measure than many other bedside echocardiographic parameters, it represents a potential target for AI-assisted automation [36].

Zhai et al. compared automated LVOT-VTI assessment by an AI tool with manual assessment by ICU physicians. In this study, 46 ICU doctors, each with more than 3 months of cardiac POCUS training, performed echocardiographic assessments of ICU patients within 2 h of admission. In addition to standard echocardiographic parameters, LVOT-VTI was measured manually and automatically using the AI tool. The automated system required an anteriorly angulated apical five-chamber view proximal to the aortic valve. The AI software classified studies as ideal, good, or unacceptable based on image quality in the region of interest, and the analysis was based on the ideal and good-quality groups. In both groups, the automated measurements showed strong correlation with manual measurements, with no significant difference between the automated and manual values [28].

From a hemodynamic standpoint, this is a compelling application because LVOT-VTI is clinically useful but is often avoided in routine bedside practice due to its technical requirements. The study suggests that AI may reduce friction in obtaining and interpreting this measurement. However, the model remained dependent on obtaining an adequate view, and its performance was evaluated among operators with some POCUS training [28]. In that regard, the data supports AI-assisted efficiency more than universal usability.

The evidence for automated LVOT-VTI measurement should also be interpreted differently from the evidence supporting automated LVEF assessment. LVOT-VTI is clinically useful, but it remains acquisition-dependent and technically more demanding. Current data support the feasibility of AI-assisted measurement in acceptable-quality studies and with trained hands, but they do not yet support its use as a universal bedside tool.

Collectively, these findings suggest that AI-assisted measurement of LVOT-VTI may improve the feasibility of technically challenging hemodynamic assessments. However, practical adoption will require validation in less selected bedside settings.

### 2.7. AI-Assisted Cardiac POCUS Education

AI may also have a role in cardiac POCUS education by supporting learners during image acquisition and improving the interpretation of focused cardiac ultrasound findings. Two recent studies evaluated this application in undergraduate and emergency medicine training settings [29,30].

Soliman-Aboumarie et al. evaluated the effect of an AI-assisted cardiac POCUS training session among undergraduate medical students. The intervention combined a brief introductory lecture with supervised hands-on practice using AI-assisted image guidance. After the session, students reported improved confidence in obtaining basic cardiac views, adjusting gain and depth, and identifying key cardiac structures [29]. These findings suggest that AI-assisted teaching may facilitate early skill acquisition and improve learner confidence during initial cardiac POCUS training. However, the study primarily assessed self-reported confidence rather than objective diagnostic performance or skill retention over time. As a result, the findings are best interpreted as supporting educational feasibility and learner acceptability rather than improved competency or lasting skill acquisition.

Dadon et al. evaluated in a randomized controlled trial the AI-assisted image guidance in clinicians working in the ED learning focused cardiac ultrasound. Following a brief didactic course, participants were randomly and equally divided into an intervention and a control group, both of which were shown echocardiography clips and asked to assess LVEF. Following each clip assessment, only the intervention group was shown the results of the AI-based tool. Both groups were then presented with a new set of 40 clips and asked to evaluate the LVEF. The study found that those using an AI-based didactic tool for the “normal–abnormal” category evaluation showed improved accuracy in consecutive clip assessments, whereas the control group showed a decline. In the “significantly reduced LVEF” category, the intervention group showed a significantly smaller decline in clip assessment than the control group. This study offers a more objective educational endpoint than learner confidence alone by evaluating short-term performance in interpreting LVEF. However, it still does not establish long-term retention, independent scanning competency, or improved clinical outcomes [30].

Taken together, these studies suggest that AI may function as a useful adjunct in cardiac POCUS education, particularly during early training and in settings with limited expert supervision. However, the current evidence should be interpreted cautiously. Improved learner confidence after AI-assisted training is not the same as objective performance improvement, and short-term gains in image interpretation do not necessarily indicate durable competency. Available studies have generally focused on learner confidence, short-term interpretation performance, or simplified classification tasks rather than independent scanning ability, long-term retention, diagnostic accuracy in real patient care, or patient-centered outcomes. Therefore, AI-assisted education should be viewed as a supplement to supervised cardiac POCUS training rather than a replacement for structured teaching, hands-on practice, expert feedback, and longitudinal competency assessment.

### 2.8. Detection of Underrecognized Cardiomyopathies

AI-assisted cardiac POCUS may help detect underrecognized cardiomyopathies, including hypertrophic cardiomyopathy and transthyretin amyloid cardiomyopathy, which are often underdiagnosed in routine clinical practice [31,37,38]. Early identification of these conditions is potentially clinically relevant, as both are associated with significant morbidity and may benefit from earlier diagnosis and targeted management.

Oikonomou et al. evaluated an AI-assisted algorithm designed to detect underrecognized cardiomyopathies using cardiac POCUS videos obtained from two large centers. The model was trained to identify hypertrophic cardiomyopathy and transthyretin amyloid cardiomyopathy and was tested across separate datasets to assess diagnostic performance. The AI model demonstrated good diagnostic performance in detecting transthyretin amyloid cardiomyopathy, with high specificity and sensitivity across both study sites. Performance was more variable in hypertrophic cardiomyopathy, with lower specificity in one cohort, suggesting that POCUS-based AI detection may be more challenging [31]. These findings indicate that AI may be able to identify specific cardiomyopathies from cardiac POCUS images, although performance may vary depending on the underlying pathology and image quality.

The clinical relevance of this application is notable. Transthyretin amyloid cardiomyopathy has increasingly been recognized as an important and often underdiagnosed cause of heart failure, particularly in older adults, with prognostic modifier interventions [37]. Hypertrophic cardiomyopathy is also frequently underdiagnosed, in part because of variable clinical presentation and limitations in routine screening [38]. AI-assisted cardiac POCUS may help identify these conditions earlier, including in patients without classic clinical features. Despite these promising findings, several limitations remain. The study did not directly compare AI performance with expert interpretation, and diagnostic accuracy was influenced by image quality. In addition, the algorithm was trained to identify a limited number of cardiomyopathies, which may restrict its applicability in broader clinical practice [31]. Overall, this is one of the more progressive applications in the field, but it remains less developed than automated LVEF assessment.

## 3. Discussion

Overall, the current evidence is strongest for AI-assisted assessment of left ventricular systolic function, particularly LVEF [17,18,19,20,21,22,23,24,32]. This is the application with the most supporting data, the clearest bedside use case, and the most direct comparison with expert interpretation or formal imaging. Other applications, including pericardial effusion detection, guidance for image acquisition, education, IVC assessment, diastolic function assessment, LVOT-VTI measurement, and cardiomyopathy detection, remain less well established [15,25,26,27,28,29,30,31,34].

Compared with automated LVEF assessment, AI-assisted diastolic assessment remains less mature. The task is more complex because it depends on multiple parameters rather than a single visual or quantitative estimate of systolic function. Unlike LVEF estimation, diastolic assessment is not a single bounded measurement and is affected by Doppler-derived parameters, loading conditions, rhythm status, and clinical context. Therefore, AI-assisted diastolic assessment should remain investigational until validated against comprehensive formal echocardiography in broader bedside populations.

Most current studies show that AI can help generate measurements, classify images, or improve short-term performance under study conditions. That is important, but it is not the same as showing that AI improves bedside decision-making, changes management, reduces diagnostic error, improves workflow, or affects patient outcomes. At present, AI-assisted cardiac POCUS is best viewed as a tool to support bedside assessment rather than as an independent diagnostic strategy.

Real-world use remains a major limitation. Many AI-assisted cardiac POCUS models have been developed or tested using selected datasets, controlled acquisition conditions, expert-acquired images, or limited operator groups. These conditions may not reflect routine ED and ICU practice. In acute care settings, patients may be unstable, obese, mechanically ventilated, poorly positioned, or difficult to scan. Operators also vary in training and experience, and ultrasound platforms vary in image quality, software integration, and workflow. As a result, performance reported in certain datasets or controlled studies may overestimate performance in routine bedside care. Broader adoption will require prospective external validation across different patients, operators, devices, and clinical environments, with outcomes that include not only agreement with expert interpretation but also workflow performance, management decisions, diagnostic error, and patient-centered outcomes.

Medicolegal accountability is another important consideration. Regulatory clearance should not be interpreted as proof that an AI tool is reliable in all bedside settings or that it can function as an independent diagnostic system. In acute care settings, AI-assisted cardiac POCUS should be treated as decision-support. Clinicians remain responsible for determining whether images are adequate, whether AI output fits the clinical context, and whether formal echocardiography or specialist consultation is needed. This is particularly important when AI tools are used by less experienced individuals or outside the populations and workflows in which they were validated. Institutions adopting these tools will need clear standards for training, documentation, quality review, and escalation when AI findings conflict with clinical judgment or image quality is limited.

Bias and external validity are also important concerns. AI-assisted cardiac POCUS models may perform differently across demographic groups, racial and ethnic populations, body habitus, comorbidities, and clinical settings. This is especially relevant if development datasets do not fully represent patients with poor acoustic windows, obesity, advanced age, critical illness, or diverse racial and ethnic backgrounds. Device variability is another major issue. Image appearance and algorithm performance may differ across ultrasound vendors, handheld platforms, probe types, acquisition protocols, and software environments. A model trained at one institution or on one device may perform less reliably when applied in another hospital, health system, or low-resource setting. These shifts may lead to performance degradation outside the development dataset. Future validation studies should include diverse patient populations, multiple institutions, different device platforms, and lower-resource environments before AI-assisted cardiac POCUS is more widely implemented.

Other technical limitations also need to be considered. AI models may be affected by dataset bias, overfitting, limited explainability, and domain shift when used outside the settings in which they were developed. In cardiac POCUS, performance may vary across ultrasound vendors, device platforms, acquisition protocols, operators, and patient populations. A model trained on selected clips from one system may perform differently when applied to handheld devices, lower-quality images, or more heterogeneous ED and ICU populations. These concerns emphasize the need for prospective external validation, transparent reporting, and post-implementation monitoring.

Another important limitation in the current literature is heterogeneity. The studies included in this review differ substantially in operator population, clinical setting, reference standard, ultrasound platform, and study endpoints. Table 2 summarizes the range in evidence-based and bedside applicability across current AI-assisted cardiac POCUS applications, showing which applications have more solid supporting data and higher immediate bedside relevance with more developed features. In general, the most well-established applications involve structured or relatively bounded tasks, whereas more complex physiologic and diagnostic applications remain less well established. Some studies evaluate agreement with expert interpretation of the same acquired TTE clip, whereas others compare AI-assisted POCUS findings with formal TTE or assess prognostic or workflow-related outcomes. As a result, these applications should not be viewed as interchangeable, and apparent diagnostic performance in one domain should not be assumed to be translated to another.

Broader technical limitations also need to be considered. AI models may be affected by dataset bias, overfitting, limited explainability, poor calibration, and domain shift when used outside the setting in which they were developed. These concerns are also emphasized in the broader AI and echocardiography literature [39,40,41,42,43,44,45,46,47,48,49,50]. In cardiac POCUS, performance may vary across patient populations, ultrasound vendors, handheld platforms, operators, acquisition protocols, and institutions. In cardiac POCUS, performance may vary across patient populations, ultrasound vendors, handheld platforms, operators, acquisition protocols, and institutions. Limited explainability may make it difficult for clinicians to understand why an AI output is incorrect, while poor calibration may lead the model to appear confident even when its reliability is low in a new clinical setting. These concerns reinforce the need for transparent reporting, prospective multicenter external validation, calibration assessment, reporting of failure modes, and post-implementation monitoring.

A further limitation is that objective performance reporting is inconsistent across studies. Some studies report sensitivity, specificity, accuracy, correlation, agreement, or analyzability, whereas others emphasize feasibility, workflow integration, learner confidence, or clinical implementation without the same level of diagnostic performance detail. For this reason, qualitative terms such as “promising” or “clinically useful” should be interpreted with caution unless supported by objective metrics and appropriate reference standards. In this review, we interpret AI performance in relation to the type of endpoint reported, including technical agreement, diagnostic accuracy, educational effect, workflow feasibility, or clinical outcome.

A major challenge in interpreting this literature is that different AI technologies are often grouped under the broad category of AI-assisted cardiac POCUS. This can be misleading. Automated LVEF assessment, acquisition guidance, education, pericardial effusion detection, cardiomyopathy classification, IVC assessment, and LVOT-VTI measurement are not equal tasks. Each has different technical requirements, different failure points, and different levels of bedside readiness. In this review, we interpreted each application separately rather than treating AI-assisted cardiac POCUS as a single clinical tool.

A related source of heterogeneity is the context in which the AI tool is evaluated. Studies using expert-acquired clips, offline interpretation, or comprehensive echocardiographic workflows should be interpreted differently from studies evaluating real-time handheld POCUS at the bedside. Similarly, acquisition guidance for novice users is a different intervention from automated interpretation of images already obtained by trained operators. These differences matter because performance in expert-acquired datasets may overestimate performance in routine ED or ICU POCUS, where operators, image quality, patient positioning, acoustic windows, and time pressure vary significantly.

The classifications in Table 2 are intended as a qualitative synthesis instead of a formal grading system. Evidence strength and practical readiness were judged based on several considerations, which included prospective versus retrospective design, presence of external or multicenter validation, diversity of patient populations and operators, use of clinically relevant reference standards, dependence on image quality and operator skill, bedside workflow feasibility, and whether the studies demonstrated clinical impact beyond technical agreement or short-term performance.

The narrative design of this review also introduces limitations. The search strategy was targeted rather than systematic, and study selection was based on author review and expert judgment rather than formal dual-review screening. In addition, the search was limited to PubMed and Google Scholar. Although these databases captured the major clinically relevant studies identified for this review, using additional databases, such as Scopus or Web of Science, may have identified additional studies and would improve comprehensiveness in future systematic reviews.

The field would also benefit from greater standardization in study design and reporting. More consistent use of reference standards, clearer definitions of operator experience, and more explicit differentiation between image acquisition support, automated quantification, and diagnostic classification would improve comparability across studies. Future studies should also better distinguish technical performance from clinical utility. Demonstrating that an AI tool can generate a measurement or classify an image with reported agreement under study conditions is not the same as showing that it improves bedside decision-making, reduces diagnostic error, changes management, or improves patient outcomes. The field would also benefit from greater standardization in study design and reporting.

**Figure 1:**
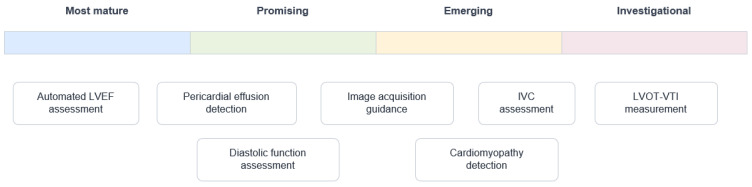
Conceptual spectrum of current artificial intelligence applications in cardiac point-of-care ultrasound. The figure summarizes major AI applications in cardiac POCUS by relative maturity and likely bedside applicability, ranging from more established uses such as automated left ventricular ejection fraction assessment to emerging or investigational applications such as IVC assessment, LVOT-VTI measurement, diastolic function assessment, and cardiomyopathy detection. The positioning is based on a qualitative synthesis of the available literature and is intended as an illustrative overview rather than a formal scoring system.

From an emergency medicine and bedside ultrasound perspective, the most likely early use cases are those in which AI helps with a relatively binary task or makes an operator-dependent measurement easier to obtain and interpret. These include automated LVEF assessment, image-guided acquisition, and potentially selected applications such as pericardial effusion detection. In contrast, more complex hemodynamic and structural applications remain promising but less established. Figure 1 illustrates a conceptual spectrum of clinical readiness. Applications were positioned based on relative technical maturity, breadth of validation, bedside feasibility, dependence on image quality and operator skill, and evidence of clinical impact. More established applications are those with multiple supportive clinical studies, clearer reference standards, and more direct bedside use cases. Emerging or investigational applications are those with narrower validation, greater technical or physiologic complexity, greater dependence on selected images or operators, or limited evidence that use changes clinical decision-making or patient outcomes.

Further prospective studies are needed to evaluate performance across larger and more diverse patient populations, assess integration into real-world workflows, and determine whether AI-assisted cardiac POCUS improves clinical decision-making and patient outcomes.

## 4. Limitations

This review has several limitations. First, this was a narrative review based on a targeted search of PubMed and Google Scholar rather than a systematic review. Study selection was based on author review and expert judgment, and no formal PRISMA workflow, duplicate screening process, or risk-of-bias assessment was performed. As a result, some relevant studies may not have been included.

Second, the included literature is heterogeneous. Studies differed in clinical setting, operator experience, ultrasound platform, acquisition context, reference standard, AI function, and study endpoint. Some studies used expert-acquired images or offline analysis, whereas others evaluated handheld or bedside POCUS use. These differences limit direct comparison across applications and make it difficult to generalize performance from one setting to another.

Third, much of the current evidence focuses on technical feasibility, agreement with expert interpretation, or short-term educational performance rather than clinical benefit. Few studies show that AI-assisted cardiac POCUS changes management, reduces diagnostic error, improves workflow, or affects patient-centered outcomes. In addition, many models remain dependent on adequate image quality and have limited external validation across diverse patients, operators, devices, and acute care environments.

Finally, broader AI-related limitations also apply, including dataset bias, overfitting, limited explainability, vendor- and device-related domain shift, and potential performance degradation outside development datasets. These issues are especially relevant in ED and ICU settings, where patients may be unstable, difficult to scan, or different from the populations used to train or validate AI models.

## 5. Conclusions

AI is beginning to play a meaningful role in cardiac POCUS, particularly in applications involving automated quantification of left ventricular systolic function and guided image acquisition. Current evidence suggests that AI-assisted cardiac POCUS may improve feasibility, standardization, and bedside assessment in selected use cases, especially when applied to focused and structured tasks.

However, most available studies evaluate technical performance, agreement with expert interpretation, or short-term educational outcomes rather than direct clinical benefit. As a result, AI-assisted cardiac POCUS is best understood as a tool to support bedside assessment rather than to replace clinician expertise or comprehensive echocardiography, and its results should be interpreted in this context. Clear clinical oversight, documentation standards, and accountability will be needed before these tools are widely integrated into acute care workflows.

## 6. Future Directions

Future work should focus on prospective multicenter external validation, clearer evidence of clinical utility, and integration into routine acute care workflows. Future studies should also report calibration, explainability where available, model failure modes, and performance across different devices, institutions, operators, and patient populations. In particular, the field needs studies that test whether AI-assisted cardiac POCUS improves decision-making, changes management, reduces diagnostic error, or affects patient-centered outcomes. These studies should include mixed ED and ICU populations, nonexpert operators, different ultrasound platforms, and clinically relevant modes such as poor acoustic windows, incomplete examinations, and unstable patients. Until then, the most appropriate role for AI-assisted cardiac POCUS is to support image acquisition, serve as a didactic tool, automate selected measurements, and assist with interpretation in focused clinical scenarios.

## Figures and Tables

**Table 1:** Representative studies of artificial intelligence applications in cardiac point-of-care ultrasound.

Study	Population/Setting	Application	AI Function	Key Findings	Major Limitations/Notes
Luong et al. [17]	Expert-acquired echocardiographic clips; offline analysis	LVEF	Automated quantification	Strong agreement with expert estimates and formal echo reports for analyzable clips	Highly image-quality dependent; only a minority of clips were analyzable by the model; Clips assessed offline.
Tromp et al. [18]	Nurse-led at-home outpatient screening	LVEF, LAVI	Quantification + screening support	Feasible AI-assisted detection of cardiac dysfunction in a pragmatic home-based model	More persuasive for screening feasibility than stand-alone diagnosis; image acquisition remained limiting; Clips assessed offline.
Dos Santos et al. [19]	Primary care patients with suspected HF	HF workup	Workflow integration	Supported earlier HF identification and treatment initiation in a primary-care pathway	Does not isolate AI effect from broader workflow changes
Cheema et al. [20]	COVID-19 ICU case series	Bedside ventricular/hemodynamic assessment	Guidance + automated measurement	Illustrated real-time bedside decision support in high-acuity patients	Very small series; illustrative rather than definitive
Dadon et al. [21]	COVID-19 patients; handheld US	LVEF	Real-time decision support	Excellent correlation with expert assessment; reduced EF predicted worse outcomes	Single clinical context; still depends on adequate acquisition
Dadon et al. [22]	Medical students performing handheld cardiac POCUS	LVEF	Decision support + novice augmentation	Substantially improved novice agreement with cardiologist and formal echo assessment	Small cohort; educational setting
Dadon et al. [23]	Follow-up registry from student-AI cohort	LVEF prognosis	Risk stratification	AI-assisted reduced systolic function predicted mortality and readmission	Registry follow-up; indirect evidence of workflow impact
Bisignani et al. [24]	Adult handheld US cohort vs cardiac MRI	LVEF	Automated quantification	Supported technical validity of handheld AI-assisted EF quantification against a higher-level reference standard	Selected cohort; external validation still needed
Yıldız Potter et al. [25]	POCUS clips with positive and negative controls	Pericardial effusion	Detection + localization	High sensitivity/specificity and accurate localization	Small dataset; broader validation needed
Blaivas et al. [26]	POCUS IVC clips reviewed by experts	IVC collapsibility	Classification	Moderate agreement with expert interpretation	Underlying metric has known clinical limitations; limited validation set
Gottlieb et al. [27]	ED physicians using handheld US	Diastolic dysfunction	Multi-parameter analysis	Good diagnostic performance for systolic and diastolic dysfunction against expert interpretation	No full TTE reference standard
Zhai et al. [28]	ICU physicians with >3 months POCUS training	LVOT-VTI	Automated quantification	Strong correlation with manual measurements in acceptable-quality studies	Requires adequate acquisition and trained operators
Soliman-Aboumarie et al. [29]	Undergraduate medical students	Education/acquisition	Acquisition guidance	Improved learner confidence for obtaining and recognizing basic cardiac views	Confidence outcome rather than diagnostic competency
Dadon et al. [30]	ED clinicians	Education/interpretation	Didactic feedback	Improved short-term LVEF interpretation performance	Pilot study; limited long-term outcomes
Oikonomou et al. [31]	Multicenter cardiac POCUS datasets	ATTR-CM, HCM	Classification	Good performance for ATTR-CM; more variable for HCM	Limited disease scope; image quality influenced performance

Abbreviations: AI, artificial intelligence; ATTR_CM, transthyretin amyoloid cardiomyopathy; ED, emergency department; HCM, hypertrophic cardiomyopathy; HF, heart failure; ICU, intensive care unit; IVC, inferior vena cava; LAVI, left atrial volume index; LVEF, left ventricular ejection fraction; LVOT-VTI, left ventricular outflow tract-velocity time integral; MRI, magnetic resonanse imaging; POCUS, point of care ultrasound; US, ultrasound.

**Table 2:** Qualitative synthesis of selected current artificial intelligence applications in cardiac point-of-care ultrasound.

Application	Evidence Strength Based on Current Validation	Bedside Readiness	Primary Limitation
LVEF	Higher	Most mature decision support application	Still dependent on analyzable image quality and broader external validation
Pericardial effusion	Moderate	Potential triage-type application	Small validation datasets and limited real-world testing
IVC assessment	Low–moderate	Standardization Tool Only	IVC collapsibility is an imperfect physiologic surrogate for volume status and fluid responsiveness
Diastolic function	Low-Moderate	Investigational	Requires multivariable Doppler-based assessment, clinical context, and stronger reference-standard validation
LVOT-VTI	Moderate in selected studies	Feasible in trained hands	Technically demanding acquisition and limited validation in less selected bedside settings
Education	Moderate for short term learning support	Practical Training Adjunct	Limited evidence for durable competency, independent scanning ability, or clinical outcomes
Cardiomyopathy detection	Early	Experimental	Limited disease scope, selected datasets, and need for broader external validation

## Data Availability

No new data were created or analyzed in this study. Data sharing is not applicable to this article.

## Abbreviations

AI	artificial intelligence
ATTR_CM	amyloid transthyretin cardiomyopathy
ED	emergency department
HCM	hypertrophic cardiomyopathy
HF	heart failure
ICU	intensive care unit
IVC	inferior vena cava
LAVI	left atrial volume index
LVEF	left ventricular ejection fraction
LVOT-VTI	left ventricular outflow tract velocity–time integral
MRI	magnetic resonance imaging
POCUS	point-of-care ultrasound
US	ultrasound
